# Microparticles That Form Immune Complexes as Modulatory Structures in Autoimmune Responses

**DOI:** 10.1155/2015/267590

**Published:** 2015-08-02

**Authors:** Catalina Burbano, Mauricio Rojas, Gloria Vásquez, Diana Castaño

**Affiliations:** ^1^Grupo de Inmunología Celular e Inmunogenética, Instituto de Investigaciones Médicas, Facultad de Medicina, Universidad de Antioquia (UdeA), Calle 70 No. 52-21, Medellín, Colombia; ^2^Unidad de Citometría de Flujo, Sede de Investigación Universitaria, Universidad de Antioquia (UdeA), Calle 70 No. 52-21, Medellín, Colombia

## Abstract

Microparticles (MPs) are induced during apoptosis, cell activation, and even “spontaneous” release. Initially MPs were considered to be inert cellular products with no biological function. However, an extensive research and functional characterization have shown that the molecular composition and the effects of MPs depend upon the cellular background and the mechanism inducing them. They possess a wide spectrum of biological effects on intercellular communication by transferring different molecules able to modulate other cells. MPs interact with their target cells through different mechanisms: membrane fusion, macropinocytosis, and receptor-mediated endocytosis. However, when MPs remain in the extracellular milieu, they undergo modifications such as citrullination, glycosylation, and partial proteolysis, among others, becoming a source of neoantigens. In rheumatoid arthritis (RA) and systemic lupus erythematosus (SLE), reports indicated elevated levels of MPs with different composition, content, and effects compared with those isolated from healthy individuals. MPs can also form immune complexes amplifying the proinflammatory response and tissue damage. Their early detection and characterization could facilitate an appropriate diagnosis optimizing the pharmacological strategies, in different diseases including cancer, infection, and autoimmunity. This review focuses on the current knowledge about MPs and their involvement in the immunopathogenesis of SLE and RA.

## 1. Introduction

It is considered that the development of any autoimmune disease requires a combination of genetic predisposition, exposure to environmental risk factors, hormones, and defects in epigenetic mechanisms that regulate immune tolerance [1]. It has been described that adaptive immunity plays a central role involving autoantibody formation, the presence and activation of autoreactive T cells, defects in regulatory functions, and the induction of anergy in these cells, among other mechanisms [2]. However, during recent years there is growing evidence regarding the participation of innate immunity in autoimmune diseases in different models. Innate immunity has an important role at the beginning of the immune response and later, perpetuating certain systemic inflammatory effects by the release of soluble factors (e.g., cytokines, chemokines and lipid mediators), the presentation of autoantigens in an inflammatory context, the activation of effector T cells, and tissue damage, among others [3].

In addition, the development of autoimmunity has been associated with defects in the pathways that regulate cell death and the recognition and clearance of apoptotic cells (ACs) [4]. Defects in the induction of apoptosis contribute to the survival of autoreactive B cells that produce autoantibodies [5]. The inefficient removal of apoptotic bodies, once they undergo posttranslational modifications in the extracellular environment such as oxidation and citrullination [6], converts them into a primary source of autoantigens, neoantigens, and immune complexes.

Microparticles (MPs) are vesicular structures mainly produced during activation and cell death; however, the precise mechanism by which they are generated is under investigation. It has been observed that MPs contain a variety of molecules inside and on the surface of them with agonist and antagonist activities; therefore, MPs can regulate the proliferation of endothelial cells [7], coagulation, thrombosis [8], inflammation, and other events related to innate and adaptive immunity. The recognition of MPs and their modification by innate immune cells could contribute to the chronic inflammatory process seen in autoimmune diseases. However, little is known about the detailed roles of MPs in the pathogenesis of these conditions [9, 10]. Only recently the number of studies relevant to the participation of these vesicular structures in the development and maintenance of autoimmune diseases such as systemic lupus erythematosus (SLE) and rheumatoid arthritis (RA) is increasing.

MPs from patients with autoimmune diseases can participate in the development of immune complexes (ICs) through interaction with circulating autoantibodies and in different tissues. Therefore, MPs can interact with target cells through different receptors such as phosphatidylserine (PS) and scavenger receptors, and they can also be recognized by opsonic receptors such as the immunoglobulin (FcR) and [11, 12] complement (CR) receptors. This opens a wide range of additional effects and potential interactions whose complexity is difficult to predict in the context of an inflammatory response.

The aim of this review is to present evidence that supports MPs and their ICs as potential immunomodulators in the context of autoimmune responses and diseases. First, some general aspects regarding the generation of and the physiological roles attributed to these structures are described. Then, the present review focuses on and discusses the potential role of MPs and their ICs in the pathophysiology of SLE and RA with respect to the promotion of inflammatory responses and tissue damage.

## 2. Definition and Overview of MPs

MPs, from different points of view, are heterogeneous structures: in size (100–1000 nm), cell origin, mechanism of induction, composition, and stability. These particles are derived from the plasma membrane of different cell types, and hence they can contain several components from the parent cell [13]. MPs were first identified in 1967 by ultracentrifugation of plasma from healthy human subjects; it was possible to obtain material rich in phospholipids with procoagulant properties. These structures were originally called “platelet dust” because it appeared to contain traces of these cells [14]; currently they are called MPs.

**Figure 1:**
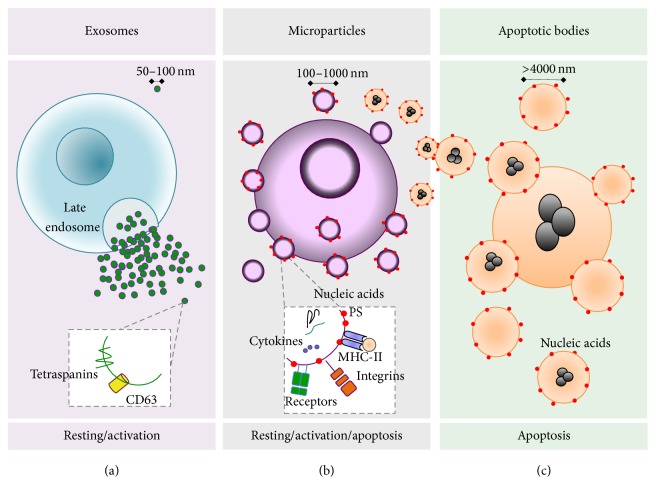
The main characteristics of secreted vesicles. (a) Cells under basal or activated states release vesicles from internal compartments such as multivesicular endosomes, also called late endosomes. Fusion among the endosomal membranes and the cell membrane leads to secretion of intravesicular bodies, which once released are called exosomes and may contain components such as TSG101 and endocytic tetraspanins (CD9 and CD63). (b) Activated cells may secrete vesicles by direct budding of the plasma membrane, called MPs, that contain various receptors, integrins, selectins, cytokines, and nucleic acids. These molecules can be located inside or on the surface of the MPs; however, a cell at rest or in response to physiological stimuli can also produce MPs, but upon activation it produces increased amounts. (c) Apoptotic cell death leads to the formation of apoptotic bodies and MPs, which may contain histones and nucleic acids. The aminophospholipid phosphatidylserine (PS) is exposed on the outer face of the cell membrane during apoptosis. MPs that express PS on their surface can also be generated by cleavage processes from apoptotic bodies.

MPs are small extracellular vesicles also known under the name of microvesicles. They are considered different from other vesicular structures such as exosomes and apoptotic bodies in size, composition, and number [15] (Figure 1). In order to differentiate MPs from other structures, they have been called ectosomes, which refers to “*bodies that emerge from the plasma membrane by ectocytosis,*” as it happens during exocytosis [15].  Table 1 summarizes the main characteristics that distinguish MPs from other vesicles.

Blood cells from mammals can generate a variety of MPs under different stimuli; however, it has been reported that approximately 80% of these circulating vesicles are derived from platelets [16]. MPs can also be generated from other cellular origins at the tissue level, for example, from tumor cells, ischemic tissue, and mesenchymal cells [13, 17]. Therefore, MPs can be found in almost any anatomical location including intercellular spaces, blood vessels, and the lymphatic system [18]. It has been reported that the plasma concentration of MPs in healthy subjects is from 5 to 50 *μ*g/mL (according to the protein content) or from 10^5^ to 10^6^ plasma membrane-derived vesicles/mL [19].

**Figure 2:**
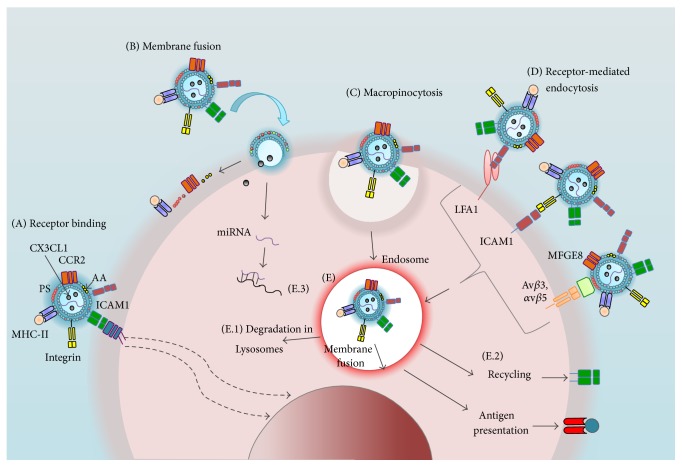
Interaction of MPs with their target cells. (A) MPs can interact with a variety of receptors on a target cell that may or may not lead to intracellular signaling (dashed arrows). Additionally, MPs can transfer their surface components (e.g., arachidonic acid (AA), PS) and internal proteins, receptors (MHC-II, CCR5), and nucleic acids (miRNA) to the target cell by (B) membrane fusion, (C) macropinocytosis, or (D) receptor-mediated endocytosis. In the latter, MPs can engage ligands such as LFA1 (lymphocyte function-associated antigen 1), intercellular adhesion molecule 1 (ICAM1), or through binding to integrins (*α*v*β*3 or *α*v*β*5) by soluble proteins that recognize the PS MFGE8 (milk fat globule EGF factor 8 protein). (E) When antigen-presenting cells internalize MPs, these structures can take different pathways: (E.1) Degradation by the endocytic pathway and subsequent antigenic peptide presentation through MHC-II molecules. (E.2) Their components may be partially recycled to the surface of the target cell, leading to a gain of phenotype and/or function. (E.3) miRNA can modulate gene expression.

The structural components of MPs include cell membrane receptors and/or glycoproteins in native or modified forms, nucleic acids (DNA and RNA), enzymes, cytokines, transcription factors, and in some cases secondary messengers (for further information review [20]). This diversity in MP content suggests that they can interact with different cells and can transfer their constituents to viable cells by different specific and nonspecific mechanisms of recognition such as membrane fusion, receptor-mediated endocytosis, and macropinocytosis (Figure 2) [21]. A recent report showed that at least platelets might contain mitochondrial structures and also release the mitochondria with proinflammatory effects [22], even though several references indicate that MPs lack complete signaling pathways and fully organized organelles [23]. The content of these structures confers them some functionality as agonists or antagonists of diverse biological processes involving intercellular communication wherein the modulatory effects of MPs are recognized. Therefore, it has been suggested that MPs may mediate pathological effects in several autoimmune diseases.

## 3. MP Generation and Components

Eukaryotic cells are constantly exposed to environmental changes and physiological stimuli that induce modifications and remodeling of the cell membrane [24], including cell division and differentiation and structural changes of the cytoskeleton during cell migration. These processes are associated with MP release [23]. Apparently, there is not an exclusive mechanism leading to the production of MPs, but it is postulated that their generation must correspond to a highly regulated process and not to a random phenomenon as it was originally suggested (reviewed in [20]).

At this point, at least two essential biological events that trigger MP generation have been described: changes in the cell membrane and changes to the cytoskeleton, both of which are dependent on intracellular calcium levels [25, 26]. However, because calcium undergoes complex regulation and is associated with multiple signaling pathways such as mechanisms of cell death and cell activation, the existence of a specific pathway for MP production remains unknown.

The composition of the lipid bilayer in the cell membrane differs between the inner and the outer sides, and it is controlled by transport enzymes that consume ATP such as “flippases” (inward lipid transport) and “floppases” (outward lipid transport) [27]. Stimuli that increase the intracellular concentration of calcium promote floppase activity, which is involved in the translocation of PS to the outer face of the cell membrane. Calcium also inhibits flippase activity, responsible for maintaining PS on the inner side of the membrane. The “scramblases” are bidirectional lipid conveyors activated by increases in the intracellular calcium levels; therefore, phospholipid changes follow their concentration gradient and they become randomly distributed in the membrane [20, 28]. Apparently, imbalance in the lipid bilayer, cytoskeletal reorganization, and proteolysis by calcium-dependent calpains lead to shrinkage of the cell membrane and MP release. In general, the exposure of PS on the outer side of the membrane appears to be frequently associated with MP release [27]; this happens transiently during cell activation and membrane remodeling [29] and permanently during apoptosis and necrosis [25, 26]. However, unknown nature annexin V− MPs have been reported [30], since some of the mechanisms by which they are released from the membrane may happen even during cell activation without PS exposure [31]. In this regard, it is considered that any stimulus that induces calcium mobilization, cytoskeletal reorganization, and cell membrane changes can induce the formation of MPs [32].

Multiple reports have indicated that apoptotic pathways, which involve the exposure of PS and the formation of blebs from the cell membrane, culminate in AC and MP formation. Apoptosis may contribute to the generation of MPs in two ways: (1) by decreasing the volume of ACs due to inactivation of ATP-dependent ionic pumps (such as Na+/K+ pump that regulates cellular water balance) and the continuous proteolysis of the cytoskeleton; (2) through the activation of ROCK-I (Rho-associated protein kinase I) by the GTPase Rho in early stages of apoptosis. This kinase regulates cortical myosin-II contraction and plasma membrane detachment of the cytoskeleton; thus, it may contribute to MP release (reviewed in [33]).

The formation of blebs during apoptosis of human neutrophils is dependent on the phosphorylation (by MLCK) of myosin light chains [34]. Apparently, most MPs are produced by this mechanism and only a few of them are released from cells by exocytotic budding; therefore, it is expected that more vesicles derive from blebbing compared to exocytic budding during apoptosis. We hypothesize that MP production during apoptosis can facilitate the disposal of dead cells; because these structures are smaller and have a compact structure, they could be removed more easily by phagocytic cells than apoptotic bodies [10, 35].

MPs generated during apoptosis may contain DNA, messenger RNA (mRNA), and microRNA (miRNA) [15]. In MPs derived from the human T cell line Jurkat or the human promyelocytic cell line HL-60 treated with staurosporine, camptothecin, or UV-B irradiation [36], the internucleosomal chromatin fragmentation usually observed in apoptosis is evident in these structures [37]. In addition, the presence of fragmented mRNA similar in size to miRNA was detected in these vesicles; however, the functional meaning of this finding is unknown beyond its potential as an autoantigen source [36]. Staining with propidium iodide and treatment with RNase and DNase have revealed the presence of surface nucleic acids on MPs. This enzymatic treatment also reduced the binding of MPs to anti-DNA antibodies [36].

There is no obvious mechanism to explain the inclusion and surface expression of nucleic acids on MPs. It is postulated that MPs generated during early apoptosis cannot possess DNA, whereas those released later during apoptosis or due to cytoskeletal and cell membrane damage could include more endogenous components (DNA, RNA, and transcription factors). This phenomenon can partially explain the heterogeneity of these structures and the reason why the evolution of MPs in the extracellular milieu might transform them into other type of structures with different effects. Proteomic analyses have shown that MP composition depends upon the culture conditions and the kind of stimuli used to induce them in addition to the cellular source [38–41]. Regarding their composition, function, and effects, the studies of Pisetsky have suggested that the most divergent types of MPs are those generated by apoptosis and cell activation [42]. Some of the biological responses induced by MPs and their association with the cellular origin and composition of these structures are summarized in Table 2. For a comprehensive review of the more common methods used for the induction of MPs* in vitro* and their implications in phenotypic changes, go to [33, 43].

## 4. MPs as an Important Source of Autoantigens

It has been proposed that excessive production of MPs (e.g., from the presence of environmental factors such as certain infectious agents and/or chronic exposure to drugs) may predispose one to autoimmune diseases [10]. Excess MPs and their wide distribution through interstitial areas could prevent their efficient clearance and allow them to become a potential source of neoantigens [12]. Neoantigens are derived from oxidative and nonoxidative modifications that mainly include citrullination, cysteine oxidation, phosphorylation, glycosylation, sumoylation (for its acronym small ubiquitin-related modifier), and covalent addition of fatty acids such as palmitoylation.

Citrullination is a posttranslational modification that converts peptidylarginine ends to peptidyl citrulline through the enzyme peptidylarginine deiminase (PAD). This posttranslational change has important implications in the pathophysiology of RA. RA patients develop autoantibodies against citrullinated peptides and/or polypeptides (anti-CCPs or ACPA) [69]. MPs are present in the synovial fluid (13) where PAD isoforms 2 and 4 are functional and the citrullination process is remarkably high [70]. Therefore, surface proteins on MPs can also become citrullinated leading to the formation of neoantigens. Cloutier et al. [12] showed that the antibodies from synovial fluid of RA patients recognize platelet-derived MPs; blockade assays indicated that this interaction is mediated at least in part by the presence of citrullinated peptides on these structures. The isolation of CD41+ MPs from the synovial fluid of RA patients by affinity columns and their further proteomic analysis showed that these vesicles have IgG specific to citrullinated peptides and C3a on their surface. Total antibodies eluted from these MPs recognize several targets such as apolipoprotein A1, citrullinated forms of clusterin, fibrinogen alpha and beta chains, vimentin, filaggrin, and histones H2A and H2B [12].

In the pathogenesis of RA and SLE, important components of MPs are generated during the apoptosis of certain types of cells including platelets and leukocytes. These components include histones and the nonhistone nuclear protein HMGB1 (high-mobility group protein1). These nuclear molecules can promote vascular and nonspecific immune responses and the generation of autoantibodies [71]. Although DNA and histones located in MPs are an important source of autoantigens, HMGB1 can trigger additional effects in host cells. HMGB1 is mainly found in the nucleus of cells, but it can be translocated to the cytoplasm and the extracellular space during cell activation and death [42]. It was observed that healthy individuals who received 2 ng of LPS/Kg of body weight showed an increase in circulating CD14+ CD42a+ MPs with surface expression of HMGB1 [72]. HMGB1 can undergo different posttranslational modifications such as cysteine oxidation; this particular change leads to its recognition by TLR4. In addition, oxidized HMGB1 can bind the chemokine CXCL12 and induce chemotaxis through CXCR4 [73]. Therefore, MPs containing oxidized forms of HMGB1 can signal through CXCR4 and TLR4. HGMB1 has been detected on the surface of MPs and as part of their ICs in the synovial fluid of RA patients [74]. In SLE, HMGB1 [42] was reported to be a component of some MPs containing anti-DNA antibodies forming ICs [74]. In summary, HMGB1 on MPs could be a key component of the immunomodulatory effects of MPs [75]; this modified protein can act as an “adjuvant” due to its ability to directly bind TLRs [72, 74].

There are other posttranslational modifications described in autoimmune diseases that generate neoantigens and could be involved in the immunomodulatory effects of MPs in these pathologies. However, they have not yet been studied in detail. For example, the oxidation and nitration of different biomolecules by peroxynitrite were described in SLE [76] and glycosylation of serum proteins was reported in RA [77]. Despite the presence of specific antibodies against these neoantigens in patients, the direct participation of these modifications on the phenotype of MPs is still unknown.

As previously mentioned, the interaction of MPs with target cells may occur by mechanisms both dependent and independent of surface receptors. Receptor-mediated mechanisms of recognizing MPs and their constituents include several members of the scavenger receptor family, PS receptors, and integrins, among others. Although the evidence is clear that MPs can interact with antibodies [78], the effects that mediate these interactions through particular receptors are not completely understood. When MPs bind to antibodies, various immune cells, mainly phagocytes through Fc receptors (FcR) and complement receptors, might recognize them. Therefore, it is tempting to postulate that MPs forming ICs, in addition to being a source of modified substrates (neoantigens) with the ability to directly bind TLRs, could amplify the effects due to the increased number of receptors with which they can interact and cross-react. In fact, it has been determined that circulating MPs from SLE patients expose PS on their surface and are coated with IgG, IgM, and C1q; these molecules are considered to be the identity signals of antibody deposition and complement activation in tissues [79]. The number of MPs binding IgG and the amount of IgG in these particles are increased in SLE patients compared to those with RA or Sjögren's syndrome (SS) [80]. Hence, it is expected that the amount and isotype of these antibodies on MPs (MP-ICs) could partly determine the means of recognition by target cells and their responses. The binding of MPs to opsonic and nonopsonic receptors could trigger complex immune responses in several autoimmune diseases, probably through the activation of multiple signaling pathways that depend on the stoichiometry of the components that constitute the MP-ICs [10].

## 5. Involvement of MPs and Their ICs in SLE and RA

Changes in the number and composition of circulating MPs have been associated with the immunopathology of different autoimmune diseases and could potentially become a diagnostic and prognostic tool.

### 5.1. MPs in SLE

In SLE patients, tissue deposition of ICs leads to chronic damage in several organs. ICs are formed mainly by autoantibodies against nuclear constituents such as double-stranded DNA (ds-DNA), nucleosomes, ribonucleoproteins, and RNA. However, in SLE there are additional autoantigens with the potential to form ICs such as phospholipids, plasma, and extracellular matrix proteins, among others [81, 82].

In patients with SLE, circulating MPs differ in their amount and composition compared to MPs from patients with other autoimmune diseases or from healthy controls. Proteomic analysis has revealed that MPs from SLE patients contain more immunoglobulins (mainly IgG (some directed against dsDNA), IgA, and IgM (anti-dsDNA, rheumatoid factor)) and complement proteins (C1q, C1s, C3, C4b, and C9) than those from healthy controls, RA patients, or SS patients [83]. SLE patients also have increased numbers of annexin V+ MPs in their plasma compared to healthy controls [84]. This increase was found to be due to platelet-derived MPs (CD41A+), which were probably activated (CD62P+). An inverse and significant correlation was found between the number of MPs with anti-dsDNA antibody and the disease activity index (measured by SLEDAI) in patients with SLE [84]. However, platelet-derived MPs are considered to be less immunogenic due to their very low to undetectable amounts of DNA [78, 85–87]. Nevertheless, it has been reported that platelet-derived MPs are capable of forming ICs and inducing complement activation [80], a typical feature of SLE pathophysiology.

It is noteworthy that apoptosis-derived MPs from different cellular sources are able to compete with ACs for PS receptor binding on mononuclear phagocytes. It was demonstrated* in vitro* that the presence of these MPs from Jurkat cells leads to a significant reduction in the phagocytosis of ACs in a dose-dependent manner; this effect was prevented by PS blockade through annexin V [19]. It has been extensively reported that the uptake and clearance of ACs by phagocytic cells are reduced in SLE patients [88]; therefore, the increase in the number of MPs observed in this disease could be a further explanation for the prolonged presence of ACs at extracellular locations in these patients.

However, there are contradictory results regarding the number of annexin V+ MPs in SLE. In 2011, Nielsen et al. reported a decreased amount of these particles in SLE patients compared to healthy controls. However, those patients had a higher frequency of annexin V− MPs mainly derived from endothelial cells, and other cells sources were not identified in that study [30]. This suggested that MP generation in SLE patients can be an event independent of apoptosis that is probably mediated by cell activation. A high amount of annexin V− MPs is positively correlated with disease activity (measured by SLEDAI), the presence of active nephritis, hypertension, arterial thrombosis, and elevated triglyceride titers [30]. In addition, the low exposure of PS by MPs in these patients could lead to decreased clearance by monocytes and macrophages. This may favor the persistence of these structures in the extracellular milieu where they can be modified by nitrosylation [89], oxidation, and citrullination [12, 90].

Ullal et al. demonstrated that MPs generated by staurosporine treatment (an apoptosis inducer) of the myeloid cell lines HL-60 and THP-1 and the CD4 T cell line Jurkat exposed histones, DNA, and nucleosomes on their surface [78]. Antibodies present in the plasma from patients with SLE bind to such* in vitro* generated MPs more strongly than antibodies from healthy controls, which display weak to absent binding activity. This interaction was not entirely affected by DNase and RNase treatment, suggesting that these antibodies might interact with other antigens [78]. The amount of surface-bound IgG is greater in circulating MPs from SLE patients than those from healthy controls. There is a significant and positive correlation between the titer of anti-DNA antibodies and the circulating amount of IgG+ MPs in SLE patients [78]. Thus, it is important to study the role of these vesicles in conjunction with their modifications and their ability to form ICs in the inflammatory processes observed in different tissues in these patients, for example, lupus nephritis.

Recently, Nielsen et al. showed an increased concentration of MPs in SLE patients with augmented levels of IgG1, IgM, and C1q compared to healthy controls. The number of circulating IgG1+ MPs was significantly associated with the presence of autoantibodies in serum against dsDNA, extractable nuclear antigen and histones [79]. Although these authors did not discuss this latter finding in detail, it is possible to speculate that the elevated number of apoptotic leukocytes observed in some patients with SLE [91] favors the generation of MPs containing nuclear components on their surface able to form ICs. In the same study, a positive correlation was found between the amount of circulating IgG+ MPs, the presence of anti-C1q antibodies (but not with other autoantibodies, such as anti-dsDNA, anti-ENA, and anti-histone), and complement consumption through the classical pathway (complement proteins C4, C3, and C1q) [79]. These results highlight the possible role of MPs in the pathogenesis and perpetuation of the inflammatory process in SLE because they constitute an important source of autoantigens and circulating ICs able to activate complement cascades.

**Figure 3:**
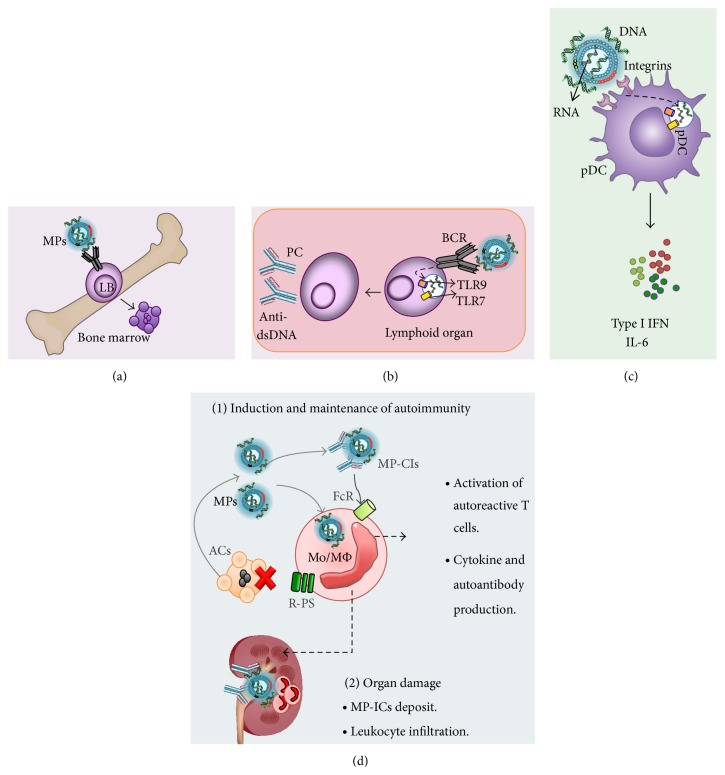
Role of MPs in SLE. MPs can interact with B cells (LB) (a) during ontogeny-induced apoptosis (clonal deletion), secondary rearrangement, or BCR edition in cells whose BCRs recognize DNA with high affinity. (b) At the lymphoid organ level, MPs can also bind to an autoreactive BCR and induce anergy of LB or alternatively be endocytosed by these cells and induce a second signal through TLR9 and TLR7 by the DNA and RNA present on these structures. These recognition activate and differentiate B cells into plasma cells able to produce autoantibodies. (c) MPs can be internalized by plasmacytoid dendritic cells (pDCs) and through the recognition of nucleic acids produce type I IFNs and other cytokines such as IL-6. (d) MPs might compete with ACs to bind PS receptors on monocytes and macrophages (Mo/MΦ), which seem to contribute to the lower uptake of ACs observed in these patients. In addition, MPs can be a major source of autoantigens in SLE with the consequent generation of ICs; all this could eventually (1) produce and maintain the inflammatory immune response and (2) promote the damage of different tissues and organs in patients with SLE due to the exacerbated inflammatory process.

Pisetsky and Lipsky [9] proposed an interesting model of the pathogenesis of MPs in SLE based on their own results and those from other authors (Figure 3). MPs that contain DNA and RNA can behave as self-adjuvants and increase tolerance of immature B-lymphocytes and break the tolerance of mature B cells. Immature B cells that recognize DNA on MPs with high avidity can be negatively selected. In contrast, self-reactive B lymphocytes that escape from central tolerance mechanisms can recognize and endocytose MPs through their BCR at the periphery. This might favor contact of the nucleic acids present on MPs with endosomal TLR9 in B lymphocytes. This interaction may trigger their activation and differentiation into plasma cells with the consequent production of autoantibodies in a manner independent of T lymphocytes.

These authors also propose that MPs endocytosed by plasmacytoid dendritic cells through integrins or PS receptors are able to contact intracellular TLR7 and TLR9, leading to cell maturation and the production of proinflammatory cytokines such as type I interferons (IFNs-I) and IL-6. The presence of these serum factors in SLE patients has been positively correlated with disease activity [92]. IFNs-I act directly on the adaptive immune response, inducing the differentiation of Th1 lymphocytes, the proliferation of memory CD8+ T cells, and the generation of plasmablasts. IL-6 mediates the differentiation and survival of the latter cells into long-lived IgG-secreting plasma cells [92]. Therefore, these cytokines produced by innate cells are involved in the autoantibody secretion and the consequent IC formation observed in this autoimmune disease [93, 94]. It is not clear what role monocytes and macrophages play in the recognition of MPs during the immunopathology of SLE. However, based on the findings reviewed herein, it could be speculated that these cells are activated by MPs and these MPs may or may not form ICs. These could be reached through Fc receptors (CD16, CD32, CD64, and TOSO), complement receptors (CRs), TLRs, and PS receptors (PSRs and “scavenger” receptors). Furthermore, MPs can compete with apoptotic cells for PS receptors, which promotes the persistence of such structures in the extracellular space, perpetuating the source of autoantigens and the inflammatory process in SLE (Figure 3).

### 5.2. MPs in RA

This disease is characterized by the presence of IgG antibodies against citrullinated proteins and rheumatoid factor (RF) in circulation and in synovial fluid. RF corresponds to an IgM antibody against the Fc portion of IgG but it can also be the IgA and IgE isotypes.

Platelet-derived MPs (PMPs) and leukocyte-derived MPs with procoagulant effects (LMPs) are increased in the circulation and synovial fluid of patients with RA [10, 84]. These MPs have been associated with disease activity, as measured by the DAS28 score, and they are also associated with joint inflammation, cartilage and bone destruction, angiogenesis, and pain [71, 95]. MPs have also been found in synovial fluid from patients with osteoarthritis, reactive arthritis, and microcrystalline arthritis; however, patients with RA and microcrystalline arthritis exhibit higher concentrations of these MPs compared with patients with osteoarthritis or reactive arthritis [96].

Leukocytes and synovial cells play critical roles in the development of joint inflammation and tissue damage and in the generation of pathogenic MPs. Synovial cells, fibroblast-like synoviocytes (FLSs), favor the development of autoimmunity because they secrete B cell activating factor (BAFF), CXCL12, and CXCL13. These factors attract B cells to the joint and promote the formation of pseudofollicles in the synovial membrane [97, 98]. Leukocytes represent the main source of MPs in the synovial fluid of RA patients, which are derived mainly from macrophages (>40%), T cells and granulocytes (20–25%), and platelets (<10%) [95]. It was reported that FLSs produce BAFF, IL-6, and IL-8 in response to MPs isolated from the synovial fluid of RA patients [96]. FLSs display elevated production of other molecules that can directly or indirectly influence the activation of B cells, such as thymic stromal lymphopoietin (TSLP) and secretory leukocyte protease inhibitor (SLPI), in response to MPs [96].

Other authors have found similar results regarding the proinflammatory effects of MPs. LMPs extracted from the joints of patients with RA induce the release of IL-6, CCL1, CCL2, and CCL5 by FLSs from the same individuals [95], and MPs obtained from Jurkat cells and U937 human promocytes induce the production of angiogenic chemokines such as CXCL1, CXCL2, CXCL3, CXCL5, and CXCL6 by FLSs from RA patients. These factors might contribute to the hypervascularization observed in inflamed joints [99]. Furthermore, it was reported that MPs can induce other proinflammatory factors such as prostaglandin E2 in FLSs [63]. This evidence suggests that MPs are actively involved in the inflammatory process in the joint and in the systemic responses observed in RA patients. Therefore, MPs function in this disease by communicating and amplifying the inflammatory response of leukocytes and other cells involved in the pathophysiology of RA.

Using flow cytometry and electron microscopy, it was revealed that MPs forming ICs (mpICs) from the synovial fluid of RA patients were larger (average size of 2800 nm) than MPs alone [12]. In these mpICs, CD41 was frequently detected suggesting that they were derived from platelets. Despite the surface detection of Fc*γ*RIIa on these particles, ICs were formed through specific recognition by autoantibodies against citrullinated vimentin and fibrinogen. In addition, these mpICs induced the production of proinflammatory leukotrienes (LTB4, 6-trans-LTB4, 12-epi-6-trans-LTB4, 20-OHLTB4, and 20-COOH-LTB4) by human neutrophils. With these sets of data, the authors suggested that MPs form circulating and articular ICs are able to induce several effects on the phagocytic cells perpetuating the inflammation. In this study, the presence of RF on MPs was not evaluated, and therefore it would be essential to determine whether mpICs from patients with RA contain this autoantibody and its implications in the immunopathology of this disease. Boilard et al. [64] reported a higher frequency of PMPs in the synovial fluid of RA patients compared with the percentage reported by Berckmans et al. [95]; apparently, these particles have an important proinflammatory role in the pathology of this disease because PMPs elicit cytokines from synovial fibroblasts via IL-1. A considerable number of patients with RA have elevated frequencies of PMPs in their synovial fluid compared to patients with osteoarthritis, in which PMPs are barely detectable [64].

Monocytes and macrophages are considered central components of the immunopathogenesis of RA. They are involved in the formation of pannus, they are one of the main producers of TNF-*α* and IL-1*β*, they participate in the activation of effector T cells, and they also have the ability to produce other cytokines and chemokines important in RA such as IL-6, IL-8, IL-10, CCL2, CCL3, and RANTES. In addition, these phagocytes are reported to be involved in the generation of autoantigens because they are a source of PAD-2 and PAD-4. Despite the central function these mononuclear phagocytes have in the immunopathology of RA and the role they must play in the recognition and clearance of MPs, it is still unknown whether these structures might induce differential effects on monocytes and macrophages depending on whether they are from patients with RA or healthy controls.

**Figure 4:**
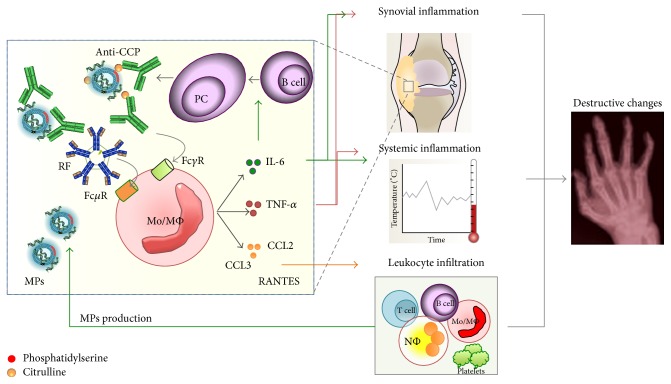
Role of MPs in RA. The high concentrations of MPs from different leukocyte populations reported in the synovial fluid from RA patients must be citrullinated and form ICs with anti-CCP antibodies and RF autoantibodies. These complex structures could be recognized by Mo/MΦ through isotype-specific Fc receptors (Fc*γ*R and Fc*μ*R) and induce the production of proinflammatory cytokines such as TNF-alpha, IL-6, and the chemokines CCL2, CCL3, and RANTES. These soluble factors participate in the systemic inflammation in the synovium and the destructive changes observed in the joints of RA patients. IL-6 is also involved in the induction of plasma cells (PCs) producing autoantibodies.

TNF-*α* has been identified as a key component of RA with multifunctional effects associated with inflammation and joint destruction [100]. The efficacy of anti-TNF-*α* treatment in RA has led to extensive research about the mechanisms that regulate its production in this disease. Reports indicate that 50 percent of RA patients are positive for anti-CCPs and circulating ICs formed by citrullinated fibrinogen [101]. These ICs induce the production of TNF-*α in vitro* by macrophages obtained from normal controls in a dose-dependent manner; this production was found to be inhibited by blockade of Fc*γ*RIIa but not Fc*γ*RI or Fc*γ*RIII [102]. In addition, the simultaneous binding of ICs containing citrullinated fibrinogen by Fc*γ*R and TLR4 induces even more TNF-*α* production by macrophages from healthy subjects [103]. Because there are increased levels of MPs forming ICs that depend on citrullinated antigens in the synovial fluid of RA patients [12], we hypothesize that the systemic inflammatory response and intrinsic activation of monocytes and synovial macrophages in RA patients may be partially explained by the recognition of these structures through Fc*γ*Rs and complement receptors. However, the potential involvement of other receptors should be noted such as Fc*μ*R (TOSO), which can recognize the FR (IgM isotype) on MPs (95) if this antibody is present in these membrane structures. Therefore, we propose that MPs could be the major source of circulating ICs in RA patients, which would lead to mononuclear phagocyte activation and the secretion of different mediators such as TNF-*α*, IL-6, and chemokines (CCL2, CCL3, and RANTES) that amplify the local and systemic inflammatory responses (Figure 4).

## 6. Conclusions and Perspectives

The evidence presented in this review indicates that MPs seem to be implicated in the autoimmune pathogenesis of SLE and RA as an important source of autoantigens and ICs. Additionally, MPs containing DNA, RNA, HMGB1, or other macromolecules could serve as adjuvants for the production of autoantibodies and perpetuate the inflammatory process in these diseases through TLR recognition. Hence, high circulating concentrations of these modified vesicular structures, forming or not forming ICs, may actively participate in the chronic inflammatory responses, severity peaks, and symptom relapses evidenced in patients with RA and SLE. However, the evidence supporting the participation of MPs in these diseases comes mainly from* in vitro* studies; therefore, advanced and improved laboratory techniques and* in vivo* experimental findings are required to allow a better understanding of the role of these structures in different contexts and in autoimmune responses.

MPs that expose PS on their surface could favor the M2 activation profile on macrophages through the binding of “*scavenger*” receptors as was previously demonstrated with apoptotic bodies. However, changes in the components of MPs (neoantigens) and interaction with autoantibodies to form ICs seem to bring these structures to phagocytic cells through other receptors that trigger M1 responses. This profile deviation in conjunction with the accumulation of MPs in circulation and different tissues could contribute to pathogenic effects on SLE and RA such as the proliferation of endothelial cells in the mitral valve [7], thrombotic events [104, 105], and complement activation, among others [86, 106–108].

Furthermore, the influence of the amount and phenotype of MPs on the activation and responses of monocyte subsets (CD14++CD16− and CD14++CD16++) have not yet been explored. It is known that CD14+CD16++ monocytes phagocytose more ACs [109] and produce more TNF-*α* in response to different stimuli. Thus, MPs may promote the response of CD14+CD16++ monocytes in RA patients, who show an increase in CD16+ monocytes [110]. In results from our group, it was observed that CD14+CD16++ monocytes were reduced in patients with active SLE [111]; this fact could reduce the removal and clearance of MPs in these patients and therefore allow an increasing source of circulating autoantigens and ICs.

It is necessary to continue the study of MPs in the context of RA and SLE and other autoimmune diseases to determine their value as biomarkers for diagnostic and prognostic purposes. For example, it should be evaluated whether certain MPs reflect a state of systemic or local activation of particular cell types or if they are associated with clinical outcome, the development of comorbidities, complications, or severe forms of RA and SLE. In addition, MPs also have attractive potential as biopharmacological agents in autoimmune diseases because they could be used in the treatment of these and other diseases as modulators of the immune response or as drug carriers to specific targets of interest.

## Figures and Tables

**Table 1:** Characteristics and properties of the main secreted vesicles.

Feature	Exosomes	Microparticles (MPs)	Apoptotic cells
Size	40–100 nm	100–1 000 nm	>4 000 nm

Coefficient of sedimentation	100 000 ×g	20 000 ×g	16 000 ×g

Methods of isolation	Sucrose gradient (1.13 y 1.19 g/mL)	Sucrose gradient, affinity column, electromagnetic sorting, and filtration	Sucrose gradient, affinity column, electromagnetic sorting, and filtration

Membrane of origin	Multivesicular endosomes	Plasma membrane	Plasma membrane

Generation	Spontaneous release and cellular activation	Spontaneous release, cellular activation, and apoptosis	Apoptosis

Annexin V binding	Low or negative	High, low, or negative	High

Functions	Carrying lytic enzymes and activation of phagocytes and B cells	Coagulation, M2 macrophage activation, and transfer of functional cell components	Antigen presentation through MHC II, M2 macrophage and monocyte activation and tissue remodeling

Markers	Rab GTPases, annexins, flotillin, Alix, TSG101, and CD63	Integrins, selectins, proteins from the parental cells, and PS	Histones, PS

Organelles	¿?	PMP might contain mitochondrial structures	Different

Nucleic acids	No	mRNA, DNA, miRNA, and interfering RNA	DNA, mRNA, and miRNA

References	[44, 45]	[22, 30, 46–50]	[51–53]

**Table 2:** MPs as mediators of communication between cells.

Effects	Cell source of MPs → target cell	*In vitro* generation of MPs	Content of MPs	REF
Chemotaxis of mononuclear phagocytes to the endothelium	Platelets → endothelial cells	Apoptosis	RANTES/CCL5	[54]

Nonspecific chemotaxis	Platelets → human neutrophils	PGE1	Fibrinogen receptor Glycoproteins and integrin *α*IIb*β*3	[33, 55]

Effects on cell proliferation and differentiation	Platelets → endothelial cells Tumoral cells (human)	Cells activation and starvation	miRNA	[56, 57]
	Platelets → endothelial precursors Tumoral cells → different types of cells	PGE1	Epidermal growth factor receptor	[33, 55]

Apoptosis of endothelial cells and inhibition of osteoclastogenesis in RA	Platelets → endothelial cells, osteoclast	Activation	miRNA-223	[58]

Induction of GLUT4 expression in insulin-resistant cells	Platelets → endothelial cells	Activation	miRNA-223	[59, 60]

Endothelial activation	Myeloid pyroptotic and activated cells → endothelium and leukocytes (humans)	Apoptosis	IL-1-*β*	[54]

Synthesis of sphingomyelinase, M2 activation, and cell death	Human macrophages → endothelial cells	Calcium-mediated apoptosis	AA	[61, 62]

Coagulation, cell transformation, and inhibition of endothelial cells	Mononuclear, endothelial, and tumoral cells → platelets	Staurosporine-induced apoptosis	Cardiolipin, platelet activating factor	[63, 64]

Coagulation	Mononuclear cells → platelets, megakaryocytes	Apoptosis	CCR5	[65]
	Monocytes → platelets (humans)	Thrombin activation	Tissue factor	[33, 55]

Antigen presentation, cross-presentation, and anergy	Among leukocytes (humans)	Apoptosis	MHC	[33, 55]

Expression of receptors from other cellular origins and transformation	Tumor, stem, and endothelial precursor cells → platelets, myeloid cell lines (humans)	Overgrowth and activation	mRNA	[66–68]
